# Spatiotemporal microvascular changes following contusive spinal cord injury

**DOI:** 10.3389/fnana.2023.1152131

**Published:** 2023-03-21

**Authors:** Nicole J. Smith, Natalie E. Doody, Kateřina Štěpánková, Martin Fuller, Ronaldo M. Ichiyama, Jessica C. F. Kwok, Stuart Egginton

**Affiliations:** ^1^School of Biomedical Sciences, University of Leeds, Leeds, United Kingdom; ^2^Centre for Reconstructive Neuroscience, Czech Academy of Sciences, Prague, Czechia; ^3^Department of Neuroscience, Second Faculty of Medicine, Charles University, Prague, Czechia; ^4^School of Molecular and Cellular Biology, University of Leeds, Leeds, United Kingdom

**Keywords:** neural trauma, angiogenesis, capillaries, stereology, regeneration, timeline, central nervous system, blood vessels

## Abstract

Microvascular integrity is disrupted following spinal cord injury (SCI) by both primary and secondary insults. Changes to neuronal structures are well documented, but little is known about how the capillaries change and recover following injury. Spatiotemporal morphological information is required to explore potential treatments targeting the microvasculature post-SCI to improve functional recovery. Sprague-Dawley rats were given a T10 moderate/severe (200 kDyn) contusion injury and were perfuse-fixed at days 2, 5, 15, and 45 post-injury. Unbiased stereology following immunohistochemistry in four areas (ventral and dorsal grey and white matter) across seven spinal segments (*n* = 4 for each group) was used to calculate microvessel density, surface area, and areal density. In intact sham spinal cords, average microvessel density across the thoracic spinal cord was: ventral grey matter: 571 ± 45 mm^−2^, dorsal grey matter: 484 ± 33 mm^−2^, ventral white matter: 90 ± 8 mm^−2^, dorsal white matter: 88 ± 7 mm^−2^. Post-SCI, acute microvascular disruption was evident, particularly at the injury epicentre, and spreading three spinal segments rostrally and caudally. Damage was most severe in grey matter at the injury epicentre (T10) and T11. Reductions in all morphological parameters (95–99% at day 2 post-SCI) implied vessel regression and/or collapse acutely. Transmission electron microscopy (TEM) revealed disturbed aspects of neurovascular unit fine structure at day 2 post-SCI (*n* = 2 per group) at T10 and T11. TEM demonstrated a more diffuse and disrupted basement membrane and wider intercellular clefts at day 2, suggesting a more permeable blood spinal cord barrier and microvessel remodelling. Some evidence of angiogenesis was seen during recovery from days 2 to 45, indicated by increased vessel density, surface area, and areal density at day 45. These novel results show that the spinal cord microvasculature is highly adaptive following SCI, even at chronic stages and up to three spinal segments from the injury epicentre. Multiple measures of gross and fine capillary structure from acute to chronic time points provide insight into microvascular remodelling post-SCI. We have identified key vascular treatment targets, namely stabilising damaged capillaries and replacing destroyed vessels, which may be used to improve functional outcomes following SCI in the future.

## Introduction

Traumatic spinal cord injury (SCI) is often a serious, life-changing injury causing sensory, motor, and autonomic dysfunction. This can manifest as, for example, hypotonia of skeletal and smooth muscle, spasticity, and pain disturbances. The primary mechanical injury followed by a secondary biochemical insult may result in long-lasting damage, not just to the neural tissue, but to all cell types within and around the injury epicentre (Ahuja et al., 2017). The severity of the secondary injury cascade can depend on the type of injury; the most common injury type is contusion with some level of compression (Dumont et al., 2001). Animal models are often used to explore aspects of human SCI, allowing control of the injury type and severity. Here, we used a standardised rat contusion model of SCI which is known to generate similar lesion and cystic cavity characteristics to human SCI (Metz et al., 2000; Cheriyan et al., 2014), and therefore often used to assess various acute and chronic aspects of the primary and secondary injury (Kjell and Olson, 2016). Understanding the physical extent of such damage and progressive changes over time may suggest new therapeutic options for people living with SCI.

The secondary injury cascade involves a plethora of factors including inflammation, haemorrhage, hypoxia, necrosis, and neurotoxicity (Mautes et al., 2000; Dumont et al., 2001; Ahuja et al., 2017). Many of these responses include damage caused to the microvasculature, or as a consequence of microvascular damage, although this important area is often overlooked. The primary mechanical injury can rupture capillaries, leading to haemorrhage and downstream hypoxia (Li et al., 2017), while disruption to the blood spinal cord barrier (BSCB) can exacerbate inflammation and necrosis of neural tissue caused by haemoglobin and haem toxicity (Bartanusz et al., 2011; Bulters et al., 2018). Collapsed or damaged capillaries may either recover or regress depending on the severity of damage to endothelial cells or other components of the BSCB, including the basement membrane, pericytes, and astrocytes (Loy et al., 2002; Simard et al., 2007; Muoio et al., 2014). This haemorrhagic and hypoxic damage can spread from the injury epicentre to form a fusiform lesion with a necrotic core (Noble and Wrathall, 1989; Simard et al., 2007), potentially leading to the cystic cavity surrounded by glial scar tissue noted in chronic SCI (Mautes et al., 2000).

Some microvascular recovery has been observed following traumatic SCI (Casella et al., 2002; Loy et al., 2002; Cao et al., 2017), although details of where and when angiogenesis (the generation of new capillaries, normally sprouting from pre-existing vessels) may occur is sparse. Angiogenesis may be observed as early as day 3 post-SCI (Popovich et al., 1996; Casella et al., 2002; Loy et al., 2002) and can occur at the injury epicentre as well as perilesional areas (Popovich et al., 1996; Loy et al., 2002). A second wave of angiogenesis may take place from days 14–60 (Popovich et al., 1996; Loy et al., 2002; Durham-Lee et al., 2012). These capillaries may not be fully functional and can regress after waves of regeneration as the new vessels do not form an effective BSCB, lacking perivascular cells such as pericytes and astrocytes (Ng et al., 2011; Cao et al., 2017). Such vessels have been shown to have a higher permeability (Whetstone et al., 2003), and so are pruned (Ng et al., 2011), leaving the injury site hypoxic as a result of capillary rarefaction.

It is currently unknown how far this capillary remodelling can spread from the injury epicentre. Capillaries will be exposed to different stimuli depending on relation to the injury epicentre, and understanding how different regions respond and recover following SCI is key to uncovering potential treatment targets. More precise morphological data are also required to differentiate between remodelling in the ventral and dorsal regions of the spinal cord. It is expected that the grey matter, with the increased cell body density and higher metabolic requirements, would have increased vessel number, vessel surface area, and areal density than the white matter under physiological conditions (Cavaglia et al., 2001; Kubíková et al., 2018; Shaw et al., 2021). This may mean that the microvasculature within the grey matter is particularly vulnerable to mechanical insults, and would therefore be a key target to repair and replace damaged perfusive vessels following injury. This study therefore aimed to create a spatiotemporal map of morphological changes to the microvasculature following SCI, analysing capillary gross and fine structure to develop novel insight into the potential for SCI regeneration therapy.

## Materials and methods

### Animals

All procedures were carried out according to the UK Home Office and local University of Leeds guidelines, in compliance with A(SP)A 1986. Twenty-four adult female Sprague-Dawley rats (200–230 g) were obtained from Charles River (Margate, UK), housed in pairs on a 12-h light-dark cycle, and fed standard chow and water ad libitum. Animals were acclimated to the facility and familiarised to handling for 7 days before surgery.

### Surgery

Rats were anaesthetised and maintained with isoflurane (5% v/v and 2% v/v, respectively, in 100% oxygen; IsoFlo^®^), and a T8/T9 laminectomy was performed. Sham laminectomy sites were packed with spongostan^TM^ before closure. For injured animals, the Infinite Horizons impactor (Precision Systems and Instrumentation, Fairfax Station, Virginia, USA) was used to deliver a 200 kDyn moderate/severe contusion injury at the T10 spinal cord level. This injury model caused complete loss of motor function below the injury level for the first 1–2 days, followed by a gradual return of weak motor function, as characterised previously (Scheff et al., 2003; Redondo Castro et al., 2011; Santos-Nogueira et al., 2012). The measured force applied by the impactor was consistent across groups (mean range: 202–204.6 kDyn). Before waking, all animals were given subcutaneous buprenorphine analgesia (Vetergesic^®^ 0.015 mg/kg) and enrofloxacin antibiotic (Baytril^®^ 2.5 mg.kg^−1^); this was continued for three days post-surgery. Bladders were manually expressed twice daily until bladder voiding ability returned.

### Spinal cord preparation

SCI rats were perfuse-fixed at days 2, 5, and 15 (*n* = 4 for all groups), and 45 (*n* = 3), with sham animals sampled at day 5 (*n* = 4). Animals were euthanised with an intraperitoneal (IP) overdose of sodium pentobarbital (200 mg/kg); IP heparin was given before transcardial perfusion with phosphate buffer (PB) followed by 4% w/v paraformaldehyde (PFA) at a flow rate of 9 ml/min. Spinal columns were post-fixed in 4% PFA at 4°C overnight. Spinal cords were dissected and cryoprotected in 30% w/v sucrose for 3 days at 4°C. The injury epicentre (T10) and three segments rostral and caudal were frozen in Optimal Cutting Temperature (OCT) mounting medium separately. 20 μm transverse sections were cut and stored on Polysine^®^ adhesion slides at −20°C.

### Immunohistochemistry

Slides were washed with phosphate buffered saline (PBS) and then blocked with 1% v/v normal donkey serum (NDS; Sigma-Aldrich, Gillingham, Dorset, UK), 1% v/v bovine serum albumin (BSA; Sigma-Aldrich), and 0.1% Triton-X in PBS. Primary antibodies for endothelial cells [1:200 mouse anti-rat endothelial cell antigen-1 (RECA-1; ab9774, Abcam, Cambridge, UK)] and basement membrane [1:200 rabbit anti-laminin (L9393; Sigma-Aldrich)] were prepared in 0.1% Triton-X PBS (PBST), and sections incubated at 4°C overnight. Secondary antibodies conjugated to Alexa-fluor 488 [1:500 goat anti-mouse (A11001; Invitrogen, Waltham, Massachusetts, USA)] and Alexa-fluor 546 [1:500 goat anti-rabbit (A11010; Invitrogen)] were also prepared in PBST. After three washes with PBS, sections were incubated with secondary antibodies for 2 hours at room temperature. Sections were washed in Tris non-saline (TNS) and mounted with Vectashield^®^ containing DAPI (Vector Laboratories, Burlingame, California, USA). Four areas (ventral grey matter, ventral white matter, dorsal grey matter, dorsal white matter) of three replicate sections were imaged for all sections at all time points and spinal segments on a Nikon E600 microscope and analysed in FIJI (NIH ImageJ; Schindelin et al., 2012).

### Stereology

Three images (318 μm × 429 μm) of replicate sections of each area were analysed for each spinal cord segment. Laminin and RECA-1 co-localisation was used to confirm vascular specificity, and RECA-1 staining was used to complete all further measurements. CD31, a commonly used endothelial marker in mouse tissue and rat peripheral tissue, was not used as it was found to have no specificity for blood vessels in rat central nervous system tissue. In FIJI (NIH ImageJ), a grid (150 μm^2^ per point) was randomly overlaid and the cell counter plugin utilised to quantify number, surface area, and cross-sectional area of vessels. Standard stereological analysis was used for vessel perimeter (boundary length, Ba = 0.5πL√d; calculated on the number of times a vessel perimeter intersected horizontal (hI) and vertical (vI) lines, where L is the average of hI and vI and d is the grid pitch (spacing of lines; 12.25 μm)) and cross-sectional area [A = dP; calculated as the product of d and number of points (P) defined by grid cross-sections lying over vessels; Weibel, 1980]. These raw values were then normalised to the area of each region of interest within an image so that vessel density was calculated as number of vessels per mm^2^, vessel surface area as vessel perimeter (μm) per mm^2^, and vessel areal density as cross-sectional area (μm^2^) per mm^2^. Surface: volume ratio (S/V; μm^−1^) was calculated from these normalised values; increased S/V implies smaller or collapsed vessels, and decreased values suggest larger, potentially less diffusive vessels. In samples where the entire region of interest was a cavity and limited tissue was present, no vessel values were inputted to avoid skewing data.

### Transmission electron microscopy

As an exploratory pilot study, animals from day 2 post-SCI (*n* = 2) and sham (*n* = 2) groups were perfused as described above with some alterations. Perfusion was carried out with 2% glutaraldehyde/2% PFA at a lower flow rate of 6 ml/min. Spinal columns were post-fixed in the glutaraldehyde-PFA mix at 4°C overnight. The injury epicentre and one segment rostral and caudal were carefully dissected with a double-edged razor. Briefly, ultra-thin sections (c. 100 nm) were cut using a diamond knife and stained with uranyl acetate (8% aqueous solution) and Reynolds lead citrate. A systematic random sampling of microvessels present in regions of interest was used to image capillaries on a T12 transmission electron microscope at an accelerating voltage of 120 kV.

### Transmission electron microscopy image analysis

Transmission electron microscopy (TEM) images taken at a magnification of 10,000× were used for capillary morphology analysis. The orthogonal thickness of the basement membrane and intercellular cleft was measured using the line tool to generate a line perpendicular to a tangent of curved objects. A minimum of four measurements distributed around individual capillaries were taken of the basement membrane and a minimum of two measurements per intercellular cleft. Intercellular cleft thickness was used to infer tight junction integrity and hence the functionality of capillaries. Due to the low number of biological replicates, a large number of technical replicates (on average 33 measurements of intercellular clefts and 53 measurements of basement membrane) per area, per group were taken and analysed to generate preliminary data in order to provide a representative overview of structural changes.

### Statistics

GraphPad Prism 9 was used for all statistical tests and graph generation. All data were first subjected to a Shapiro-Wilk test of normality. Two-way ANOVAs with multiple comparisons and a *post-hoc* Tukey test were conducted on stereology data and are reported as [F(df interaction, df within) = (F-value), *p* = (*p*-value)]. TEM data were analysed using a Kruskal-Wallis test with *post-hoc* Dunn’s test and are reported as [H(df) = (H-value), *p* = (*p*-value)]. All data are presented as mean ± standard deviation (SD) in bar charts with individual values plotted and as trend lines in line graphs.

## Results

### Microvessel density decreases post-SCI in the grey matter

**Figure 1:**
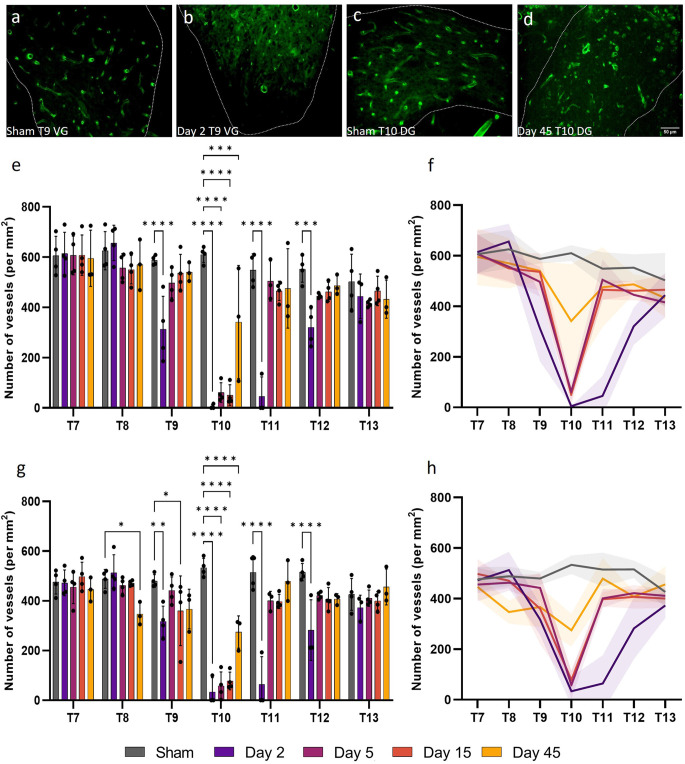
Vessel density (the number of vessels per mm^2^) in the grey matter significantly decreased following spinal cord injury. Microvessel density [visualised by RECA-1 staining (green)] significantly reduced in the ventral grey (VG) matter of T9 at day 2 (*P* < 0.0001) **(B)** compared to sham cords at the same level **(A)**. At day 45 at T10, vessel density also significantly decreased in the dorsal grey (DG) matter (*P* < 0.0001) **(D)** compared to sham **(C)**. Vascular rarefaction was significant up to two spinal segments away from the injury epicentre at T10 in both the ventral **(E,F)** and dorsal **(G,H)** grey matter. Trend lines **(F,H)** show the pattern of changes over time across seven spinal segments. Scale bar = 50 μm **(A–D)**. *P < 0.05, **P < 0.01, ***P < 0.001, ****P < 0.0001. Error bars ± SD. *n* = 3/4 for all groups.

Stereology was performed to assess changes in microvessel density at days 2, 5, 15, and 45 following a T10 moderate/severe spinal cord contusion in rats. Microvascular density was assessed as the number of vessels per unit area (in mm^2^) and can indicate the propensity of angiogenesis or regression. Statistically significant reductions in microvessel density (number of vessels mm^−2^) were observed in both the ventral and dorsal grey matter of T9, T10, T11, and T12 at day 2 after injury compared to sham values (*F*_(24, 96)_ = 7.965, *P* < 0.0001 and *F*_(24, 96)_ = 7.655, *P* < 0.0001 respectively; Figure 1). Microvessel density decreased by 99.2% in the ventral grey matter and 95.3% in the dorsal grey matter of T10 at day 2 post-SCI (*P* < 0.0001; Figures 1E,G). Microvessel density was significantly lower at day 45 in the dorsal grey matter of T8 (Day 45: 347.04 ± 45.96 vessels mm^−2^; Sham: 488.11 ± 38.11 vessels mm^−2^, *P* = 0.021; Figures 1D,E). Whilst lower capillary density in the ventral and dorsal grey matter was evident at T9, T11, and T12, particularly caudal to the injury site, this was not significant from day 5 onwards (all values *P* ≥ 0.051) except at day 15 in the dorsal grey matter of T9 (*P* = 0.043). When averaged across all segments, intact sham thoracic spinal cords had 571 ± 45 vessels mm^−2^ in the ventral grey matter and 484 ± 33 vessels mm^−2^ in the dorsal grey matter.

### Vessel surface area in the grey matter is compromised following injury

**Figure 2:**
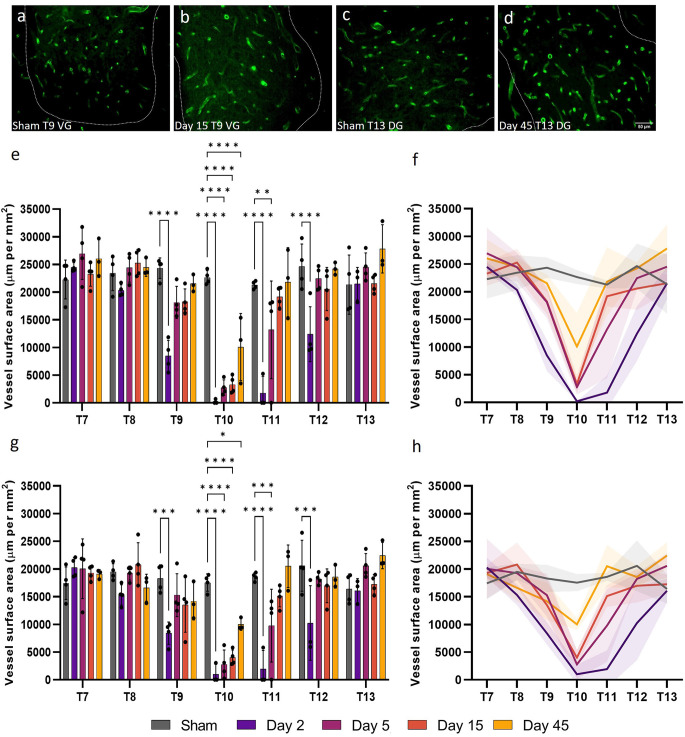
Widespread microvessel surface area disruption occurred following spinal cord injury. Vessel surface area [visualised by RECA-1 staining (green)] reduced until day 15 in the ventral grey (VG) matter at T9 **(B)** compared to shams **(A)**. An increasing trend in vessel surface area was observed at day 45 at T13 in the dorsal grey (DG) matter **(D)** compared to shams **(C)**. Spatiotemporal changes in the surface area of vessels were present as far as two spinal segments from the injury epicentre in the ventral grey matter **(E,F)** and the dorsal grey matter **(G,H)**. Trend lines **(F,H)** show the pattern of changes over time across seven spinal segments. Scale bar = 50 μm **(A–D)**. *P < 0.05, **P < 0.01, ***P < 0.001, ****P < 0.0001. Error bars ± SD. *n* = 3/4 for all groups.

Vessel surface area is a measurement of the anatomical boundary, and therefore diffusive capacity, of capillaries so can be used to gain insight into the functionality of microvessels. Consistent with reduced microvessel density, microvessel surface area (perimeter normalised for sample area) also significantly decreased at day 2 post-SCI for T9, T10, T11, and T12 in both the ventral and dorsal grey matter (*F*_(24, 96)_ = 7.593, *P* < 0.0001 and *F*_(24, 96)_ = 4.805, *P* < 0.0001 respectively; Figure 2). Microvessel surface area did not return to sham levels until day 15 in the ventral and dorsal grey matter at T11, remaining significantly lower at day 5 in both areas (Sham ventral grey: 21,287.42 ± 714.23 μm mm^−2^; Day 5 ventral grey: 13,183.44 ± 8819.67 μm mm^−2^, *P* = 0.0083; Sham dorsal grey: 18,606.12 ± 739.16 μm mm^−2^; Day 5 dorsal grey: 9772.81 ± 6571.83 μm mm^−2^, *P* = 0.035; Figure 2E). Microvessel surface area remained significantly reduced at the injury epicentre (T10) in both the ventral and dorsal grey matter at all time points (all values *P* ≤ 0.014; Figures 2E,G). There was an increase in vessel surface area at day 45 at T13 in the ventral grey matter (Day 45: 27,824.54 ± 4349.84 μm mm^−2^; Sham: 21,357.51 ± 5357.26 μm mm^−2^, *P* = 0.04) consistent with visual observations at the same time point in the dorsal grey matter (Figures 2D,E).

### SCI disrupts vessel areal density in the grey matter

**Figure 3:**
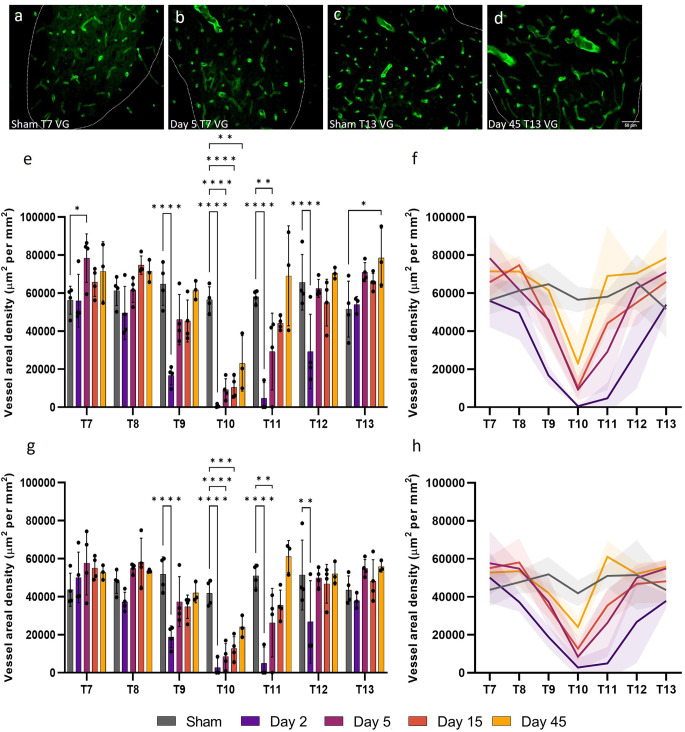
Vessel areal density was significantly compromised after spinal cord injury. At day 5, microvessel areal density increased (*P* < 0.05) **(B)** compared to shams **(A)** in the ventral grey (VG) matter of T7 [visualised by RECA-1 staining (green)]. A similar increase was observed at T13 in the ventral grey matter at day 45 (*P* < 0.05) **(D)** compared to shams **(C)**. Microvessel areal density significantly reduced at day 2 at T9, T10, T11, and T12 in both the ventral **(E,F)** and dorsal **(G,H)** grey matter. Trend lines **(F,H)** show the pattern of changes over time across seven spinal segments. Scale bar = 50 μm **(A–D)**. *P < 0.05, **P < 0.01, ***P < 0.001, ****P < 0.0001. Error bars ± SD. *n* = 3/4 for all groups.

Microvessel areal density (cross-sectional area normalised for sample area) can also be used to assess capillary functionality by providing an index of the instantaneous volumetric capacity of the microcirculation. Areal density was significantly altered up to 12 mm, or three spinal segments, both rostral and caudal to the injury epicentre (ventral grey matter: *F*_(24, 96)_ = 4.919, *P* < 0.0001; dorsal grey matter, *F*_(24, 96)_ = 3.236, *P* < 0.0001; Figure 3). As expected, at day 2, microvessels in the ventral and dorsal grey matter at T9, T10, T11, and T12 had significantly reduced areal density (all values *P* ≤ 0.0093). As areal density increased towards sham levels by day 5 at T9 and T12 (all values *P* ≥ 0.12; Figures 3E,G), this significant decrease at day 2 may be indicative of microvessel collapse. Microvessel areal density at day 45 in the dorsal grey matter at T10 was not significantly different from sham levels (Sham: 41,877.22 ± 6814.81 μm^2^ mm^−2^; Day 45: 24,118.59 ± 5795.65 μm^2^ mm^−2^, *P* = 0.12; Figure 3G). Interestingly, significant increases in vessel areal density were seen in the ventral grey matter of T7 at day 5 (Day 5: 78,410.96 ± 12,711.09 μm^2^ mm^−2^; Sham: 56,311.83 ± 7332.36 μm^2^ mm^−2^, *P* = 0.041) and of T13 at day 45 (Day 45: 78,563.98 ± 15,557.61 μm^2^ mm^−2^; Sham: 51,584.54 ± 14,673.47 μm^2^ mm^−2^, *P* = 0.014; Figures 3B,D,E); this was not observed in the corresponding dorsal grey matter (Figure 3G).

### Surface:volume ratio in the grey matter increases acutely after injury

**Figure 4:**
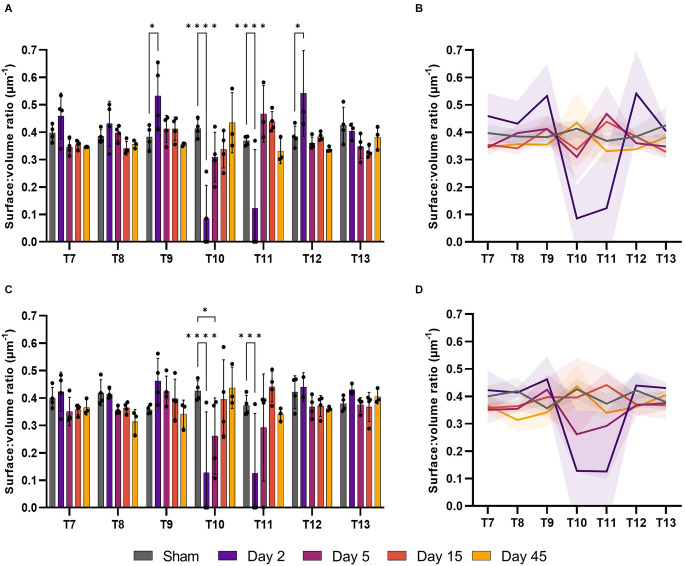
The surface:volume ratio (S/V) was disrupted after spinal cord injury. Microvessel S/V increased at day 2 in the ventral grey matter of T9 and T12 (*P* < 0.05) relative to sham operated controls **(A)**. S/V decreased at the injury epicentre (T10) at day 2 in the ventral grey matter (**A**, *P* < 0.0001) and at days 2 and 5 in the dorsal grey matter (**C**, *P* < 0.0001 and *P* < 0.05 respectively). Trend lines show the pattern of changes over time across seven spinal segments in the ventral **(B)** and dorsal **(D)** grey matter. *P < 0.05, ***P < 0.001, ****P < 0.0001. Error bars ± SD. *n* = 3/4 for all groups.

Surface:volume ratio (S/V) can be used to assess whether microvessel recovery is isotropic, or dependent on orientation/location. This measurement can give an indication of angiogenesis or regression, with values closer to 0.5 indicating a round profile and that vessels are cut transversely, while values > 0.5 may indicate collapsed vessels or those sectioned obliquely. Changes in microvessel S/V were evident in both the ventral and dorsal grey matter following SCI (*F*_(24, 94)_ = 6.055, *P* < 0.0001 and *F*_(24, 94)_ = 4.106, *P* < 0.0001, respectively; Figure 4). Microvessel S/V increased at T9 at day 2 in the ventral grey matter (39.3%, *P* = 0.017), returning towards sham levels by day 5 (Figure 4A). This effect was also observed in the ventral grey matter of T12 at day 2 (41%, *P* = 0.023). Vessel S/V significantly reduced at day 2 in both the ventral and dorsal grey matter at the injury epicentre (T10), and remained reduced at day 5 in the dorsal grey matter (Sham: 0.41 ± 0.03 μm^−1^; Day 2: 0.09 ± 0.12 μm^−1^; Day 5: 0.31 ± 0.09 μm^−1^; Figure 4C).

### Microvessel morphology in white matter is altered at chronic time points following SCI

**Figure 5:**
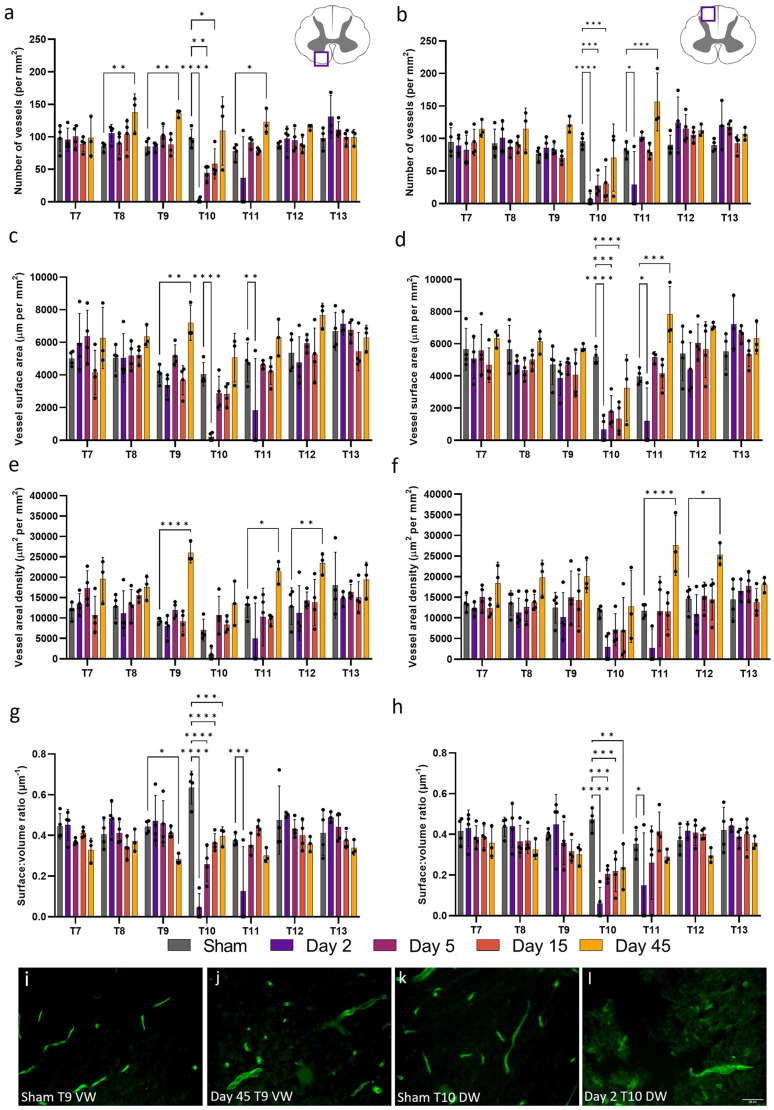
Microvessel morphology was significantly altered 45 days after spinal cord injury in the white matter. Vessel density **(A,B)**, surface area **(C,D)**, and areal density **(E,F)** were used to assess microvascular changes following spinal cord injury in the ventral **(A,C,E)** and dorsal **(B,D,F)** white matter. Vessel surface:volume ratio remained stable across most segments in the ventral **(G)** and dorsal **(H)** white matter, although was significantly reduced at day 45 post-injury at T9 (*P* < 0.05) in the ventral white (VW) matter **(J)** compared to shams **(I)**. Most microvessel measurements were significantly reduced at day 2 in the dorsal white (DW) matter **(L)** compared to shams **(K)**. *P < 0.05, **P < 0.01, ***P < 0.001, ****P < 0.0001. Error bars ± SD. *n* = 3/4 for all groups.

White matter microvessel density was 16.9% that of the grey matter microvessel density in sham operated animals, but was similar in the ventral and dorsal white matter across the thoracic spinal cord (90 ± 8 mm^−2^ and 88 ± 7 mm^−2^ respectively). Microvessel density decreased at day 2 in the injury epicentre (T10) and did not recover until day 45 in both the ventral and dorsal white matter (*F*_(24, 98)_ = 4.376, *P* < 0.0001 and *F*_(24, 98)_ = 4.433, *P* < 0.0001 respectively; Figure 5). A similar pattern was seen in other measurements [microvessel surface area (ventral white matter: *F*_(24, 98)_ = 2.453, *P* = 0.0011; dorsal white matter: *F*_(24, 98)_ = 2.890, *P* = 0.0001] and areal density [ventral white matter: *F*_(24, 98)_ = 1.643, *P* = 0.047; dorsal white matter: *F*_(24, 98)_ = 1.565, *P* = 0.066)] at day 2 in the injury epicentre, although vessel areal density was not significantly different to sham values in the ventral or dorsal white matter (Sham ventral white: 7051.28 ± 2664.61 μm^2^ mm^−2^; Day 2 ventral white: 1042.27 ± 1447.78 μm^2^ mm^−2^, *P* = 0.23; Sham dorsal white: 11,630.04 ± 1246.68 μm^2^ mm^−2^; Day 2 dorsal white: 2945.67 ± 3412.39 μm^2^ mm^−2^, *P* = 0.053; Figures 5E,F). Interestingly, significant increases in microvessel parameters were seen at day 45 in a number of segments. For example, microvessel density, surface area, and areal density all increased at day 45 in the ventral white matter at T9 (60.2%, *P* = 0.008; 77.3%, *P* = 0.0045; and 178.4%, *P* < 0.0001 respectively; Figures 5A,C,E).

### Ultrastructural damage is severe at acute time points following SCI

**Figure 6:**
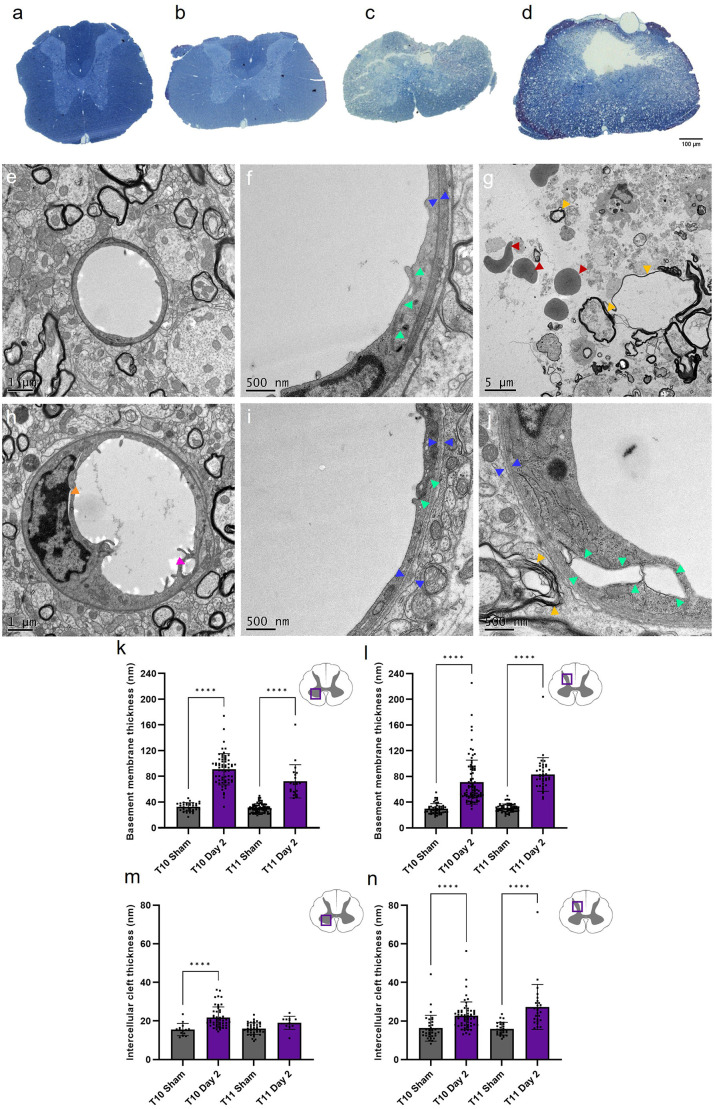
Transmission electron microscopy (TEM) revealed severe capillary damage at acute time points following spinal cord injury. Semi-thin sections demonstrated the extent of injury damage at day 2 at T10 **(C)** and T11 **(D)** compared to sham at T10 **(A)** and T11 **(B)** . Sham capillaries **(E,F)** demonstrated smooth capillary walls with narrow intercellular clefts (green arrowheads) and basement membrane (blue arrowheads) thickness. At day 2, intracellular vesicles were observed (**H**, pink arrowhead), with increased basement membrane and intercellular cleft thickness **(I,J)**. Observable parenchyma damage included cavities (white spaces), haemorrhage (red arrowheads), unravelling myelin (yellow arrowheads), and cell debris (indistinct grey structures) **(G)**. Basement membrane thickness was noticeably greater at day 2 in the ventral **(K)** and dorsal grey matter **(L)**, as was intercellular cleft thickness [ventral grey matter **(M)**, dorsal grey matter **(N)**]. Scale bars 100 μm **(A–D)**, 1 μm **(E,H)**, 500 nm **(F,I,J)**, 5 μm **(G)**. Individual points represent individual measurements of the basement membrane **(K,L)** or intercellular cleft **(M,N)** thickness. ****P < 0.0001. Error bars ± SD. *n* = 2 for all groups.

Structural changes in the neurovascular unit provide insight into microvessel regression and angiogenesis. TEM analysis of grey matter capillaries identified key indications of microvascular damage at day 2 (Figure 6). Extensive areas of necrotic cell debris were observed (Figure 6G), with large intracellular vesicles (pink arrowhead) and nuclear detachment (orange arrowhead) present in endothelial cells of grey matter capillaries after SCI (Figure 6H). The ventral and dorsal grey matter displayed significant increases in thickness in both the basement membrane (*H*_(3)_ = 139.7, *P* < 0.0001 and *H*_(3)_ = 163.6, *P* < 0.0001 respectively; Figures 6K,L) and intercellular cleft (*H*_(3)_ = 41.75, *P* < 0.0001 and *H*_(3)_ = 54.85, *P* < 0.0001 respectively; Figures 6M,N). Basement membrane thickness and electron density diffusivity increased significantly at day 2 in the injury epicentre (ventral grey matter: 91.08 ± 24.34 nm; dorsal grey matter 71.21 ± 34.22 nm) compared to sham cords (34.28 ± 6.65 nm and 29.71 ± 7.99 nm respectively, all values *P* < 0.0001), implying basement membrane degradation and therefore increased permeability of the BSCB. The same effect was seen at T11 in both regions (all values *P* < 0.0001; Figures 6K,L). Intercellular cleft thickness increased significantly following injury at T10 in the ventral (Sham: 15.52 ± 3.17 nm; Day 2: 21.91 ± 5.41 nm, *P* < 0.0001) and dorsal grey matter (Sham: 16.28 ± 6.71 nm; Day 2: 22.69 ± 7.21 nm, *P* < 0.0001). There was also a 70.5% increase in intercellular cleft thickness at T11 in the dorsal grey matter (Sham: 16.00 ± 3.29 nm; Day 2: 27.28 ± 11.65 nm, *P* < 0.0001; Figure 6N).

## Discussion

This study aimed to quantify key structural changes in capillary morphology and microstructure from the acute to chronic stages of SCI. By taking multiple time point measurements of microvessels and using unbiased stereology principles, an accurate picture has been built up of key read-outs. The microvascular pathology of SCI provides crucial insights into the overall mechanisms of injury and repair in the spinal cord. These spatiotemporal changes may help identify novel microvascular targets for SCI neuroregeneration, enabling a more holistic therapeutic strategy by combining multiple approaches.

**Figure 7:**
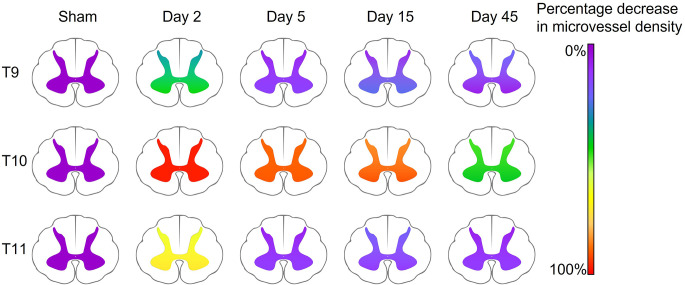
Summary schematic demonstrating the percentage decrease in microvessel density (number of vessels mm^−2^) in the ventral and dorsal grey matter compared to sham animals in key spinal cord segments. The injury epicentre (T10) demonstrated the most severe changes, with a 99% and 95% decrease in vessel density at day 2, and a 44% and 49% decrease at day 45 (ventral and dorsal grey matter respectively). Microvessel density decreased at day 2 in perilesional areas (47% and 34% at T9, and 75% and 62% at T11, ventral and dorsal grey matter respectively), however returned to only a <25% reduction in microvessel density by day 5.

Regression or collapse of microvessels at acute stages was evidenced by simultaneous reductions in vessel density, surface area, and areal density measurements compared to sham operated controls in both the ventral and dorsal grey matter. The most notable changes were located within 8 mm, or two spinal segments, of the injury epicentre (T8-T12; Figure 7). However, significant increases in vessel surface area and areal density were seen as far as 12 mm, or three spinal segments, from the injury epicentre at multiple time points in the grey matter. This indicates that vascular damage and potential dysfunction are widespread and may not recover, even after 45 days. A decrease in vessel surface area implies regression or collapse of microvessels, with the consequential reduction in diffusive capacity that may promote hypoxic cell dysfunction and/or impede trauma repair. In most segments, this decrease was transient and returned to within sham values by day 5, suggesting capillary collapse at day 2 and subsequent reperfusion at day 5 without sustained endothelial cell damage (Figure 2). However, at T9 in the ventral grey matter, this decrease was still present at day 15 post-SCI. This may indicate vessel regression, often linked with hypoperfusion consistent with traumatic vessel collapse, with subsequent angiogenesis between days 15 and 45 potentially triggered by the consequential hypoxia (Figure 7). Microvascular areal density increased in the ventral grey matter at day 5 three segments rostral to the epicentre (Figure 3) which, with microvascular regression reducing vessel number (rarefaction), may imply transient vessel oedema or dilation associated with greater individual cross-sectional area. The similar increase in areal density seen at day 45 in T13 is consistent with capillary arteriolisation as there was an increasing trend before this chronic time point. However, observational findings corroborated by large data variance indicate there is great variability between animals at day 45 across all measurements, particularly in the ventral grey matter at T10, implying angiogenesis in some animals and rarefaction in others.

An increase in microvessel areal density without a simultaneous increase in numerical density and surface area is indicative of more non-diffusive vessels rather than angiogenesis. Therefore, the S/V may provide a useful measure of gross changes in the microvasculature. An increase in S/V, as seen at day 2 at T9 and T12 in the ventral grey matter, and T9 in the dorsal grey matter, is consistent with more collapsed vessels following contusion induced hypoperfusion (Figure 4). By contrast, a decrease in S/V, as seen in the T8 dorsal grey matter at day 45, shows vessels were dilated with a larger areal density and smaller relative surface area, consistent with either passive (downstream congestion) or active (adaptive remodelling) responses to SCI. These outcomes are important to interpret together. For example, most variability is seen in the ventral grey matter, away from the more severely damaged dorsal grey matter, suggesting a dominance of passive vs. active responses respectively.

Similar, although less widespread, changes were seen in the ventral and dorsal white matter at acute time points. Significant reductions in vessel density, surface area, and areal density in both the ventral and dorsal white matter were observed at day 2, with only changes in areal density at T10 in the ventral white matter lacking significance (Figure 5). These changes corresponded with a decrease in vessel S/V at all time points following injury at T10, indicating that only larger vessels may survive at the injury epicentre (Figures 5G,H). Increases in all measurements were seen at T9 in the ventral white matter at day 45 post-SCI, potentially indicating angiogenesis. However, S/V was also significantly reduced at this time point, implying microvessel changes may not be isotropic, and therefore these vessels may not be fully functional. Increases in microvascular measurements were seen at day 45 from T7 to T12, indicating that widespread microvascular restructuring continues at chronic time points following SCI, particularly in the ventral white matter. A similar finding has been reported previously, however only spreading one spinal segment rostrally and caudally in the ventral white matter between days 28 and 60 post-SCI (Loy et al., 2002). The differences in microvascular damage seen between the grey and white matter suggest that white matter may be more resilient to SCI. This may reflect the lower metabolic requirements of the white matter, although sparing of the white matter has been linked to improved locomotor recovery (Slomnicki et al., 2020). It is also possible the white matter may possess a greater degree of hypoxia tolerance or suffer from less disruption in perfusion caused by larger vessel compression than the grey matter. Angiogenic potential may be inversely proportional to metabolic rate, meaning the grey matter may require a greater angiogenic stimulus to elicit a similar response to that seen in the white matter.

These gross structural responses were complemented by exploratory TEM investigations to elucidate accompanying microstructural changes to the microvasculature and investigate possible mechanisms relevant for future treatment options. Analysis of capillary fine structure at day 2 of T10 and T11 in the ventral and dorsal grey matter was targeted as these locations were the most severely affected by injury (Figure 6). Clear signs of endothelial damage were observed, including nuclear detachment, basement membrane swelling, intercellular cleft widening, and intracellular vesicle formation. Apparent pericyte detachment was also noted, adding to previous observations that some pericyte subtypes migrate from capillaries to form a vital part of the lesion scar (Goritz et al., 2011). Widespread tissue disruption at the epicentre was evident, where cavities contained cell debris, erythrocytes, and unravelling myelin. At day 2, both basement membrane and intercellular cleft thickness were significantly greater compared to sham values (Figures 6K–N), demonstrating an early onset of BSCB disruption. These observations have previously been noted in a model of chronic non-traumatic SCI (Xu et al., 2017), but have not been directly studied in models of acute traumatic SCI before now. These initial findings may indicate the BSCB is not fully intact at acute time points following SCI, likely contributing to the secondary injury. Previous studies in other models of CNS injury support this proposition by using Evans blue dye or dextran leakage from the vasculature into the surrounding tissue to demonstrate BSCB dysfunction (Figley et al., 2014; Nahirney et al., 2016; Xu et al., 2019). The current study did not directly study astrocyte contribution to the BSCB following SCI, however previous data show unusually limited association between neovasculature and astrocytes acutely following SCI, which may also contribute to the increased BSCB permeability (Casella et al., 2002; Ng et al., 2011). Therefore, concomitant quantification of astrocyte and endothelial cell morphology would be of benefit to give more conclusive answers about the origins of BSCB disruption. Stabilising tight junctions between endothelial cells and promoting cohesiveness of the neurovascular unit may be a potential treatment strategy to limit the damage caused by the secondary injury and provide a more homeostatic microenvironment for neuroregeneration.

Stereology is commonly used to quantify structural responses to physiological challenges by generating three-dimensional information from two-dimensional images (Howard and Reed, 1998; West, 2012). The grid overlay and unbiased sampling approach facilitate multiple measurements with a low risk of skewing data. Significant background staining and unavoidable artefacts caused by free erythrocytes and scar tissue were evident after SCI; for this reason, a manual approach was chosen to prevent inaccurate thresholding with automated image processing leading to unreliable results. Automated image analysis also demonstrates no greater statistical power than stereology, and stereology is often cited as the “gold standard” for precise, unbiased measurements (Howard and Reed, 1998; Muhlfeld et al., 2010; Evanko et al., 2018). This approach also allows comparison of the data obtained with those from other pathologies. Of particular interest is a comparison with diabetes; a disease often cited as demonstrating the worst vascular damage across multiple tissues (Tsilibary, 2003; Kolluru et al., 2012). Similar signs of vascular damage are seen in diabetes as seen here in spinal cords 45 days after injury. Parallel increases in surface area and areal density at this chronic stage in the grey matter imply an increased number of less-diffusive vessels such as dilated capillaries and/or arterioles. An increase in microvessel density may not necessarily be a positive outcome, as seen with similar changes in diabetes (Tsilibary, 2003; Adamska et al., 2019); if these vessels do not improve their diffusive capacity, the hypoxic microenvironment in the cord after SCI may not be improved.

Prior to this study, there was little information regarding spatiotemporal changes in the microvasculature following traumatic SCI. Whilst a similar contusion model was used to assess vascular changes from days 1–60 post-SCI, and also used RECA-1 staining, the majority of the analysis used laminin to demonstrate potential angiogenesis (Loy et al., 2002). Laminin is a marker of the basement membrane, and thus reflects the abluminal surface of vessels, meaning that results may be skewed by vessels appearing larger than the endothelial layer, and therefore overestimating perfusive capacity. Laminin also forms streamers following both capillary regression and angiogenesis (Krum et al., 1991; Loy et al., 2002), and as TEM demonstrated in this study, basement membrane thickness increases after injury, further adding to the potential for unreliable results of vessel measurements. However, analysis of RECA-1 stained vessels in this study validates the previous finding that stained microvessels decreased in number at acute time points and gradually increased thereafter close to the injury epicentre. Blood vessel diameter also significantly increased at chronic time points 15 mm from the injury epicentre (Loy et al., 2002), suggesting that adaptive remodelling was widespread; results corroborated in the present study.

The current study fulfilled the aim of providing important data on the morphological changes of the microvasculature following SCI, however, further research is required to build on these results to inform future treatment strategies. Primarily, co-staining for other aspects of the neurovascular unit could be of benefit, as mentioned previously, alongside markers of tight junction integrity. Previous studies have shown decreases in the expression of tight junction proteins following SCI, such as zonula-occludens-1 and claudin-5 (Benton et al., 2008; Luo et al., 2022), however incorporating this analysis alongside the more extensive spatiotemporal data shown here may give more mechanistic information into the intercellular cleft widening observed in this study. SMI-71, a marker for mature vessels previously shown to increase only in the white matter 60 days post-SCI (Loy et al., 2002), or Ki67, a marker of proliferation, could also be utilised to confirm the potential angiogenesis seen here using morphological measurements. The parameters explored in this study were chosen for their sensitivity and reliability to indicate regression or angiogenesis, however three-dimensional lightsheet microscopy may be able to offer further analysis opportunities to assess tortuosity or branching of vessels, for example. Although this study is the first to use TEM to quantify vascular morphological changes acutely following traumatic SCI, further TEM analysis could also be carried out to investigate mechanistic changes at additional time points following SCI. Future research should focus on assessing wider aspects of neurovascular unit morphology following SCI and how they may contribute to remodelling and functionality of the microvasculature at various time points post-SCI.

## Conclusions

This study has demonstrated significant microvascular damage following traumatic SCI, spreading as far as three spinal segments rostrally and caudally from the epicentre. Some microvascular recovery was observed over time following disruption, such as increased surface area and areal density, apparent up to 45 days post-SCI in the grey matter. Angiogenesis was evident during recovery, particularly in the ventral white matter, while significant variability between individuals at day 45 may indicate heterogeneity of vascular response to injury. Fine structure of aspects of the neurovascular unit following traumatic SCI demonstrated severe damage in different areas of the spinal cord at day 2 post-injury, indicating greater permeability of the BSCB and microvascular regression, but also the potential for early angiogenesis following injury to rescue some lost capillaries. The novel spatiotemporal read-outs of capillary morphology and fine microstructure following SCI from acute to chronic stages may help identify vascular targets for neuroregeneration. Further research is needed to better understand how these microvascular changes may affect recovery and inform potential treatment options to repair and replace damaged vessels. Promoting a favourable microenvironment for neural recovery in combination with established treatments may improve functional recovery following SCI.

### Transparency, rigor, and reproducibility summary

The study was not formally registered, but the analysis plan was devised and pre-specified before data collection. A sample size of four rats per group (IHC) and two rats per group (TEM) was planned based on animal availability and compliance with the 3Rs (A(SP)A). Six rats received sham surgery, and 19 rats received a T10 contusion injury. 1 rat was excluded for technical reasons, making the final groups analysed *n* = 4 for immunohistochemistry (1 further rat was excluded from the day 45 group due to observed discrepancies in the injury characteristics) and *n* = 2 for TEM. No deaths or unexpected trauma occurred during the study. Animals were randomly assigned to groups before surgery by a second experimenter. Specificity of antibodies was confirmed and optimised in previous trails using intact rat spinal cord tissue. Analysis of experimental materials was performed by investigators who were aware of relevant group information, however analysis was completed in a random order to reduce counting bias. Surgical procedures were completed over the course of multiple days. Bladder expression, cage changes, and analgesia and antibiotic injections were delivered at the same time each day/week throughout the study. Data analysis performed by a second assessor was randomly cross-checked by the primary assessor to ensure continuity. Samples collected as part of the study are available to be used for future research; please contact Nicole J. Smith.

## Data availability statement

The original contributions presented in the study are included in the article, further inquiries can be directed to the corresponding author.

## Ethics statement

The animal study was reviewed and approved by UK home office under A(SP)A.

## Author contributions

NS, ND, RI, JK, and SE conceived and/or designed the study and data interpretation. NS and ND performed all surgical and tissue collection procedures. NS performed the experiments. NS and KŠ analysed data. NS and MF collected TEM data. All authors contributed to the article and approved the submitted version.
